# Restoration of miR-193a expression is tumor-suppressive in *MYC* amplified Group 3 medulloblastoma

**DOI:** 10.1186/s40478-020-00942-5

**Published:** 2020-05-14

**Authors:** Harish Shrikrishna Bharambe, Annada Joshi, Kedar Yogi, Sadaf Kazi, Neelam Vishwanath Shirsat

**Affiliations:** 1 Advanced Centre for Treatment, Research & Education in Cancer, Tata Memorial Centre, Kharghar, Navi Mumbai, 410210 India; 2 Homi Bhabha National Institute, Training School Complex, Anushakti Nagar, Mumbai, 400085 India

**Keywords:** MiR-193a, MYC, MAX, Medulloblastoma, Promoter methylation

## Abstract

Medulloblastoma, a highly malignant pediatric brain tumor, consists of four molecular subgroups, namely WNT, SHH, Group 3, and Group 4. The expression of miR-193a, a WNT subgroup-specific microRNA, was found to be induced by MYC, an oncogenic target of the canonical WNT signaling. MiR-193a is not expressed in Group 3 medulloblastomas, despite MYC expression, as a result of promoter hypermethylation. Restoration of miR-193a expression in the *MYC* amplified Group 3 medulloblastoma cells resulted in inhibition of growth, tumorigenicity, and an increase in radiation sensitivity. *MAX*, S*TMN1*, and *DCAF7* were identified as novel targets of miR-193a. MiR-193a mediated downregulation of MAX could suppress MYC activity since it is an obligate hetero-dimerization partner of MYC. MYC induced expression of miR-193a, therefore, seems to act as a feedback inhibitor of MYC signaling. The expression of miR-193a resulted in widespread repression of gene expression that included not only several cell cycle regulators, WNT, NOTCH signaling genes, and those encoding DNA replication machinery, but also several chromatin modifiers like *SWI/SNF* family genes and histone-encoding genes. MiR-193a expression brought about a reduction in the global levels of H3K4me3, H3K27ac, the histone marks of active chromatin, and an increase in the levels of H3K27me3, a repressive chromatin mark. In cancer cells having high MYC expression, MYC brings about transcriptional amplification of all active genes apart from the induction of its target genes. MiR-193a, on the other hand, brought about global repression of gene expression. Therefore, miR-193a has therapeutic potential in the treatment of not only Group 3 medulloblastomas but possibly other MYC overexpressing aggressive cancers as well.

## Introduction

Medulloblastoma is the single most common malignant brain tumor in children [1]. Standard treatment of medulloblastoma includes surgery, followed by craniospinal radiation and chemotherapy. The treatment results in the long-term survival of about 70% of average-risk patients. However, these children often suffer from neuroendocrine and neurocognitive deficits. Medulloblastomas belong to four distinct molecular subgroups WNT, SHH, Group 3, and Group 4 that were first identified based on the differential gene expression profile [2]. Activation of the canonical WNT and SHH signaling pathway is a characteristic of the WNT, and SHH subgroup medulloblastomas, respectively. The two non-WNT, non-SHH subgroups are characterized by several chromosomal level copy number alterations, including isochromosome 17q in more than 50% tumors and have an overlap in their gene expression profile. The Group 4 tumors express several neuronal differentiation-related genes, while Group 3 medulloblastomas have higher expression of proliferation-related genes and often retina-specific genes. Recently, three studies based on the integrated genomic analysis have identified multiple subtypes within Group 3/Group 4 subgroups, which are enriched in specific driver genetic alterations and copy number variations [3–5]. A meta-analysis of the three studies identified eight subtypes with some subtypes consisting of both Group 3 and Group 4 tumors, indicating a broad continuum of the subtypes [6]. Subtypes II, III, and V have the worst five-year overall survival and often carry amplification in the *MYC* and *MYC/MYCN* gene, respectively.

Among the four subgroups, the WNT subgroup has an excellent long term survival rate of over 90% [7]. The canonical WNT signaling is known to mediate the stem cell self-renewal as well as the epithelial-mesenchymal transition, the characteristics known to be associated with aggressive cancers [8]. Nonetheless, the WNT subgroup medulloblastomas have the best survival rates among the four subgroups. We have earlier reported the most distinctive microRNA profile of WNT subgroup medulloblastomas [9, 10]. MiR-193a-3p (hereafter referred to as miR-193a) is one of the WNT subgroup-specific microRNAs [10]. MYC, an oncogenic target of the canonical WNT signaling, was found to induce miR-193a expression. MYC overexpression with or without amplification is a marker for poor prognosis in Group 3 medulloblastomas [1]. The methylation status of the CpG island in the miR-193a promoter region was studied to decipher the mechanism underlying the lack of miR-193a expression in most Group 3 tumors. The effect of restoration of miR-193a expression on the malignant behavior of the Group 3 cell lines carrying MYC amplification/overexpression was studied in detail.

## Materials and methods

### Cell culture

Dr. D. Bigner (Duke University Medical Centre, Durham, NC, USA) kindly provided the D425 medulloblastoma cell line [11]. A recently developed Group 3 medulloblastoma cell line, HD-MB03, was kindly provided by Dr. Till Milde (The German Cancer Research Center, Heidelberg, Germany) [12]. Medulloblastoma cell lines Daoy and D283 were obtained from the American Type Culture Collection, MA, USA [13]. The cell lines were validated for their subgroup status by the real-time RT-PCR analysis of the genes specifically overexpressed in the medulloblastoma subgroups [10]. The human embryonic kidney cell line HEK293FT was procured from the Thermo Fisher Scientific (Waltham, MA, USA). All the cell lines used were validated by the short tandem repeat marker profiling and tested for the mycoplasma-free status. The cell lines were maintained in the Dulbecco’s Modified Eagle Medium (D-MEM) or D-MEM/F12 medium containing 10% fetal calf serum (Thermo Fischer, Waltham, MA, USA) in a humidified CO_2_ incubator.

### Plasmid/lentiviral constructs and analysis of miR-193a promoter activity by the luciferase reporter assay

The sequences of the primers used for PCR amplification, cloning, and RT-PCR are given in Supplementary Table 1. MiR-193a encoding genomic region was amplified by the polymerase chain reaction (PCR) from normal peripheral human lymphocyte DNA. The 564 bp PCR product was cloned in the lentiviral vector pTRIPZ (Dharmacon, Lafayette, CO, USA) downstream of the doxycycline-inducible minimal cytomegalovirus (CMV) promoter. It was also cloned in the pcDNA4 myc-His B vector (Thermo Fisher Scientific, Waltham, MA, USA) for expressing miR-193a in the HEK293FT cells. MiR-193a promoter region (− 1292 bp to − 158 bp upstream of pre-miR-193a) was amplified by PCR from normal lymphocyte genomic DNA [14]. It was cloned upstream of the luciferase cDNA in the promoter-less pGL3-Basic vector (Promega Corporation, Madison, WI, USA) (Supplementary Table 1). This construct was used for evaluating the activity of miR-193a promoter by the luciferase reporter assay upon transient transfection into the HEK293FT cells. MYC binding site in the miR-193a promoter construct was altered by the site-directed mutagenesis using the overlap extension PCR, as described [15]. MYC cDNA, from the pBS-MYC expression vector (kindly provided by Dr. Joan Massague, Memorial Sloan Kettering Cancer Centre, NY, USA), was cloned in the pcDNA 3.0 vector for transient transfection into the HEK293FT cells. The expression levels of miR-193a and *MYC* were studied using the Taqman real-time RT-PCR assay (Applied Biosystems, Thermo Fischer Scientific, Waltham, MA, USA) using *RNU48* and *GAPDH* as house-keeping genes, respectively, as described before [10]. The expression levels were expressed as Relative Quantity (RQ) = 2 ^^- (Ct^_test_^-Ct^_Control_^)^ X 100.

### Methylation status of the CpG island in the miR-193a promoter region and treatment of the medulloblastoma cells using a DNA methylation inhibitor

The expression profile data and the methylation profile data (GSE85218) of 763 medulloblastomas from the MAGIC cohort [4] were retrieved from the NCBI (https://www.ncbi.nlm.nih.gov) web site. The data were analyzed for the expression levels of miR-193a and methylation status of the CpG island in the miR-193a promoter region in the four molecular subgroups of medulloblastomas. The methylation status of the miR-193a promoter CpG island in the established medulloblastoma cell lines was studied by the methylation-specific PCR following bisulfite-conversion of their genomic DNA using the EZ DNA methylation Gold kit (Zymo Research, Irvine, CA, USA). The primers for the methylation-specific PCR were used as described before (Supplementary Table 1) [16]. Medulloblastoma cells were treated with the DNA methylation inhibitor, 5-aza-2′-deoxycytidine (Sigma-Aldrich, St Louis, MO, USA) at concentrations ranging from 50 nM to 500 nM and the expression levels of miR-193a were evaluated by real-time RT-PCR assay as described before.

### Effect of miR-193a expression on medulloblastoma cell proliferation, radiation sensitivity, and cell cycle analysis

Group 3 medulloblastoma cell lines were transduced with lentiviral particles carrying the pTRIPZ-miR-193a construct. Multiple stably transduced polyclonal populations (P1, P2) were selected in the presence of puromycin. As a vector control, the cells were transduced with the parental pTRIPZ lentiviral vector. For the induction of miR-193a expression, the cells were treated with 4 μg/ml doxycycline for 48 h and the expression levels of miR-193a were evaluated by the real-time RT-PCR analysis. The effect of miR-193a expression on the growth and radiation sensitivity of the medulloblastoma cells was studied by the MTT assay as described [17]. The medium containing doxycycline was replenished at 2-day intervals. The cells were irradiated at a dose ranging from 2 Gy to 6 Gy using the Bhabhatron, a telecobalt machine developed by the Bhabha Atomic Research Centre, India, which uses Cobalt-60 isotope as a source of gamma radiation. The D_0_ dose, the dose of the radiation required to decrease the surviving fraction of cells to 37%, was calculated from the survival graph [18]. For the cell cycle analysis, medulloblastoma cells were treated with doxycycline for 96 h, then fixed in chilled 70% ethanol and stained with Propidium iodide [19]. The flow cytometry analysis was done using the Attune NxT Flow cytometer (Thermo Fisher Scientific, Waltham, MA, USA), and the data were analyzed for the distribution of the cells in different phases of the cell cycle using the ModFit analysis software.

### Effect of miR-193a expression on the anchorage-independent growth and in vivo tumorigenicity of medulloblastoma cells

The soft agar colony formation assay was used for studying the anchorage-independent growth of medulloblastoma cells. Polyclonal populations (P1, P2) of the medulloblastoma cell lines expressing miR-193a and those expressing vector control (VC) were treated with 4 μg/ml doxycycline for 48 h before seeding for the soft agar assay. One thousand cells were plated in 0.3% agar containing medium in a 35 mm tissue culture plate over a basal layer of 1% agar in complete medium containing doxycycline. One hundred fifty microliter of the medium was added to the soft agar plate at 2-day intervals. The colonies consisting of at least 20 to 25 cells were counted after 2–3 weeks of incubation. Medulloblastoma cells were orthotopically injected in the cerebellar region of the mouse brain to study the effect of miR-193a expression on tumorigenicity, as described [20]. The study was approved by the Institutional Animal Ethics Committee, and the experiments were conducted as per the ethical guidelines. NOD/SCID (NOD.CB17-Prkdc_scid_ /NCrCrl; The Jackson Laboratory, Charles River, USA) strain was used for the study. Medulloblastoma cells were transduced with lentiviral particles carrying firefly luciferase FL2 cDNA (a gift from Snorri Thorgeirsson, Addgene plasmid #55764) in order to monitor the tumor growth by in vivo bioluminescence imaging [21]. The mice were orthotopically injected with the medulloblastoma cell lines carrying either the pTRIPZ vector or pTRIPZ-miR-193a construct following treatment with doxycycline for 48 h. The cells were injected through a 0.5 mm burr hole in the skull, 2 mm posterior to lambda at 2 mm depth using a Hamilton syringe with the aid of a small animal stereotactic apparatus (Stoelting, IL, USA). The mice were administered 1 g per liter of doxycycline in a 5% sucrose solution to maintain the induction of miR-193a expression during the tumorigenicity experiments. The mice were monitored at regular intervals by in vivo bioluminescence imaging using the IVIS Spectrum (Perkin-Elmer, MA, USA) imaging system. The photon output from the image with peak luciferase activity was quantified by manually drawing the region of interest around the luminescent area and was expressed as average radiance (photons/sec/cm^2^/steridian). Kaplan Meier survival analysis was done based on the survival duration until the mice were sacrificed upon more than 15% loss of body weight or on development of clinical signs like vertigo, hunched posture, abnormal gait. Log Rank test was used to determine the statistical significance of the difference in the survival curves of the vector control and miR-193a expressing tumor-bearing mice.

### Transcriptome sequencing and identification of biological pathways significantly enriched in the genes downregulated upon miR-193a expression

Total RNA was extracted from doxycycline-treated P1, P2 populations expressing miR-193a, the parental cells, and the vector control HD-MB03 medulloblastoma cells. Illumina TruSeq RNA library preparation kit was used for preparing libraries from the Poly-A enriched RNA fraction of the total RNA. The libraries were sequenced on an Illumina Hiseq 2500 system (San Diego, CA, USA) to get a minimum of 10 million single-end reads per library. The sequence data were aligned to the reference human genome using the HISAT2 aligner software (https://ccb.jhu.edu/software/hisat2) using the default parameters, and the count of the number of reads per gene was derived using the HTSeq-count algorithm (www.bioinformatics.babraham.ac.uk). The genes significantly differentially expressed upon miR-193a expression were identified using the DESeq2 R Bioconductor package (https://bioconductor.org/packages/release/bioc/html/DESeq2.html). The gene set enrichment analysis (GSEA; http://software.broadinstitute.org/gsea/) was performed to identify biological pathways significantly enriched in the genes downregulated upon miR-193a expression. ClueGO version 2.5.5 (http://apps.cytoscape.org/apps/cluego), a Cytoscape plug-in software, was used to derive the protein-protein interaction network of the pathways significantly enriched (KEGG and the Reactome pathway databases) in the 459 genes downregulated upon miR-193a expression. Only the differentially expressed genes were used for building the network. The statistical test used in the ClueGO analysis for the enrichment of biological pathways was the Two-sided hypergeometric test, with the *p*-value cutoff of 0.01 using the Bonferroni step down as the correction method, 2% as the minimum percentage of interacting genes and the Kappa score threshold of 0.47 [22].

### Identification of the genes targeted by miR-193a in medulloblastoma cells

The genes significantly downregulated upon expression of miR-193a in medulloblastoma cells were analyzed for the presence of a miR-193a binding site in their 3′-untranslated regions (3′-UTRs) using the TargetScan software (www.targetscan.org). 3′-UTRs of known miR-193a targets *ERBB4*, *KMT2A,* and that of the putative novel targets *MAX*, *DCAF7*, *STMN1,* and *MAP3K3* were cloned downstream of the firefly luciferase cDNA in the pcDNA 3.0 vector (Thermo Fischer Scientific, Waltham, MA, USA) and the reporter activity was assayed as described [17]. The miR-193a binding sites in the 3′-UTRs were altered by the site-directed mutagenesis using the overlap extension PCR as described [15]. The luciferase reporter constructs were transfected in the HEK293FT cells along with the Green Fluorescent Protein (GFP) expressing vector as a normalization control, with or without the miR-193a expressing construct. The luciferase activity was evaluated 72 h post-transfection using the Cytation 5 Hybrid Multi-mode reader (BioTek, Winooski, VT, USA). The real-time RT-PCR analysis or the western blot analysis was used for evaluating change in the expression levels of known and novel miR-193a targets upon expression of miR-193a in medulloblastoma cells. The western blots were developed using the WesternBright ECL HRP substrate (Advansta, San Jose, CA, USA), and the images were acquired and analyzed using the Chemidoc gel imaging system (Bio-Rad, Hercules, CA, USA).

#### Following antibodies were used for the analysis

Anti-CCND1 antibody (sc-450), anti-GAPDH antibody (sc-32233), anti-p16 (CDKN2A) antibody (sc-468), anti-MYC antibody (sc-764) from the Santa Cruz Biotechnology, Dallas, TX, USA.

Anti-MCL1 antibody (PAC615Hu01) from the Cloud Clone Corporation, Wuhan, China.

Anti-MAX antibody (#4739), anti-Histone H3 (trimethyl K4) antibody (#9751), anti-Histone H3 (acetyl K27) antibody (#8173) from the Cell Signaling Technology, Boston, MA, USA.

Anti-H3 (trimethyl K27) antibody (ab6002), anti-Histone H3 (ab10799) antibody from the Abcam, Cambridge, UK.

Anti-γ-tubulin antibody (T3559) from the Sigma Aldrich, St. Louis, MO, USA.

All the experiments were performed at least three times, and the student’s t-test or the analysis of variance test was used for statistical analysis in the GraphPad Prism version 6.0 software (www. graphpad.com). Error bars indicate standard error of the mean.

### Data availability

The datasets used in the present study are available on reasonable request.

## Results

### MYC driven upregulation of promoter activity and expression levels of miR-193a, a WNT subgroup-specific microRNA

**Fig. 1:**
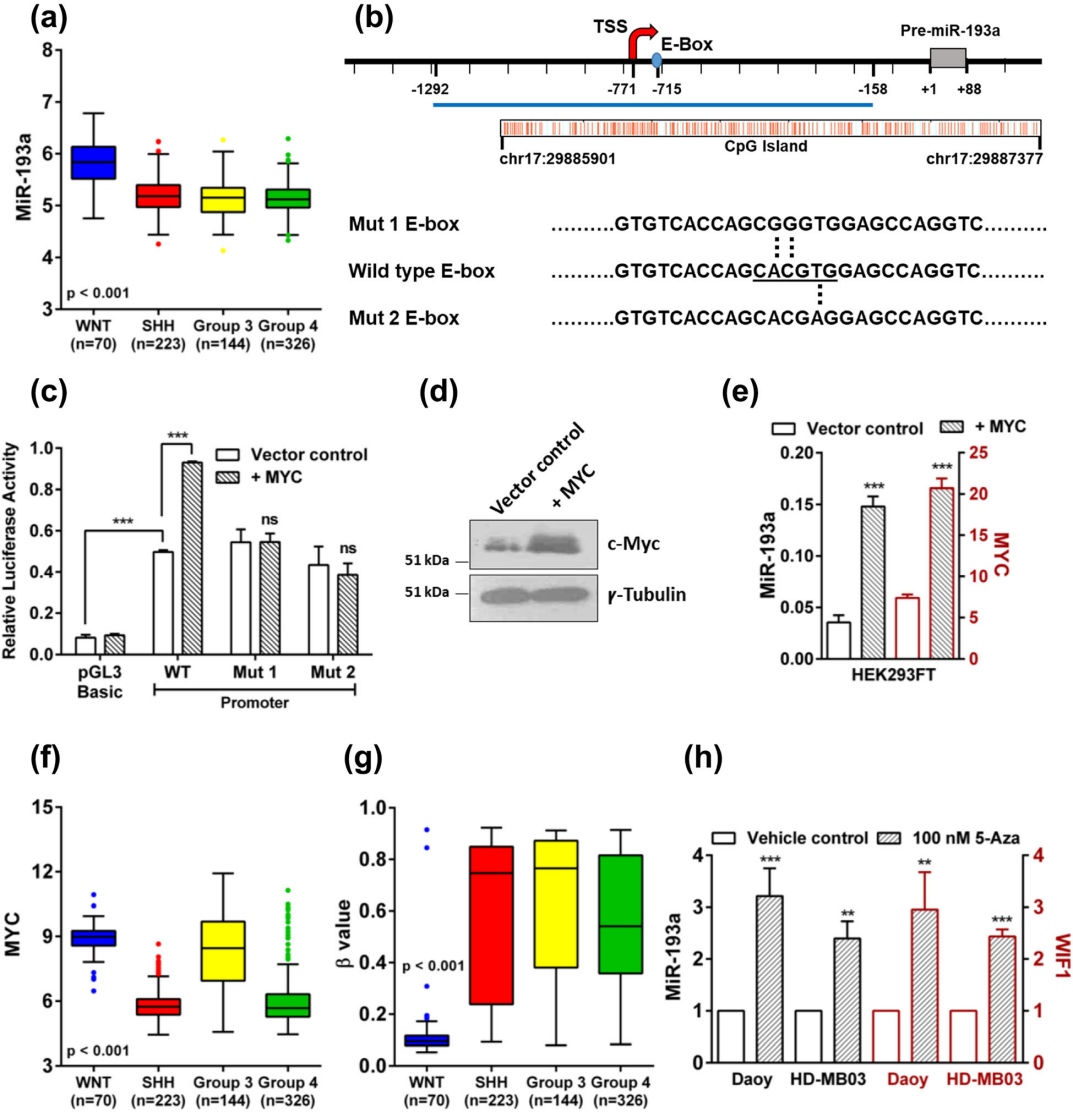
Expression of miR-193a, MYC, and the methylation status of the miR-193a promoter region, in the four molecular subgroups of medulloblastomas. Induction of miR-193a expression by MYC, and upon treatment with a DNA methylation inhibitor in medulloblastoma cells. **a** MiR-193a expression levels in the four molecular subgroups WNT, SHH, Group 3, and Group 4 of 763 medulloblastomas from the MAGIC cohort. **b** Schematic showing location of the CpG island, E-box, the transcription start site (TSS) relative to the pre-miR-193a start site (+ 1) on chromosome 17 and the mutations introduced in the E-box. **c** Relative luciferase reporter activity of the miR-193a promoter construct in the presence or absence of MYC and upon the site-directed mutagenesis of the MYC binding site in the miR-193a promoter constructs (Mut 1, Mut 2), upon transient transfection into the HEK293FT cells. **d** Western blot analysis showing MYC expression in the HEK293FT cells transfected with the MYC expressing plasmid construct. γ-tubulin was used as a loading control. **e** Induction of miR-193a and MYC expression in the HEK293FT cells transfected with the MYC expressing construct evaluated by real-time RT-PCR analysis. **f** and **g**. Expression levels of *MYC* and the methylation status of a CpG probe (cg22536383) in the miR-193a promoter region, in the four molecular subgroups of medulloblastomas from the MAGIC cohort, respectively. Higher β values indicate higher methylation at the CpG residue. **h** Fold change in the expression levels of miR-193a and *WIF1* in the 5-aza-2′-deoxycytidine treated medulloblastoma cells evaluated by the real-time RT-PCR assay. **, *** and ns indicates *p* < 0.001, *p* < 0.0001 and non-significant, respectively

We have earlier reported the differential expression of several microRNAs in the four molecular subgroups of medulloblastomas [10]. Real-time RT-PCR analysis of expression levels of miR-193a in an Indian cohort of 103 medulloblastomas showed the median expression level in the WNT subgroup medulloblastomas to be RQ = 7.5 (CI 3.3 to 19.5) (Supplementary Fig. 1A). The median expression levels of miR-193a in the SHH, Group 3, and Group 4 tumors, on the other hand, were 0.13, 0.4, and 0.09, respectively (Supplementary Fig. 1A) [10]. Analysis of the expression levels of miR-193a in the published expression profiling data of 763 medulloblastomas from the MAGIC cohort [4] also showed that expression of miR-193a is restricted to the WNT subgroup (Fig. 1a). In order to delineate the molecular basis of the WNT subgroup-specific miR-193a expression, the miR-193a promoter region was analyzed for the binding sites of the WNT signaling target transcription factors. UCSC genome browser (https://genome.ucsc.edu) showed a consensus MYC binding motif called ‘E-box’ in the miR-193a promoter region (Fig. 1b). Binding of MYC to this site in the miR-193a promoter region has been demonstrated experimentally in multiple cell lines including, K562, a myeloid leukemia cell line, and A549, a lung adenocarcinoma cell line in the ‘ENCODE’ project (www.encodeproject.org) (Fig. 1b). MiR-193a promoter region was cloned upstream of the luciferase cDNA in a promoter-less pGL3-Basic vector. Luciferase reporter assay showed a basal promoter activity of this construct upon transient transfection into the HEK293FT cells. Cotransfection with a MYC expressing plasmid construct resulted in 1.7 to 2 fold induction of the basal activity of the miR-193a promoter (Fig. 1c, d). The site-directed mutagenesis of the E-box nucleotide sequence resulted in a significant reduction in the induction of the miR-193a promoter activity by MYC (Fig. 1b, c). Furthermore, transient transfection of MYC expressing construct in the HEK293FT cells upregulated the endogenous expression levels of miR-193a by ~ 3 fold (Fig. 1d, e). The MYC-driven induction of the promoter activity and the expression levels of miR-193a suggests that MYC could induce miR-193a expression in the WNT subgroup medulloblastomas.

### Hypermethylation of the miR-193a promoter region in non-WNT medulloblastomas and restoration of miR-193a expression upon treatment with a DNA methylation inhibitor

Several Group 3 medulloblastomas and the established Group 3 cell lines overexpress MYC with or without amplification of the *MYC* oncogene (Fig. 1f, Supplementary Fig. 1B). However, the majority of these tumors lack miR-193a expression. Analysis of the MAGIC cohort [4] data for the methylation of the CpG island in the promoter region of miR-193a showed methylation of multiple CpG residues in the three non-WNT subgroups of medulloblastomas (Fig. 1g, Supplementary Fig. 1C). Thus, miR-193a expression appears to be repressed in the non-WNT tumors as a result of promoter hypermethylation. MiR-193a expression was also found to be low in the MYC overexpressing Group 3 cell lines D283, D425, and HD-MB03 [13] (Supplementary Fig. 1A). The CpG island in the miR-193a promoter region was found to be methylated in the Group 3 cell lines as well as in the Daoy cell line belonging to the SHH subgroup as assessed by the methylation-specific PCR assay (Supplementary Fig. 1D). *WIF1* is known to be silenced in these medulloblastoma cell lines as a result of promoter methylation. The expression levels of *WIF1* increased upon treatment of the cells with a DNA methylation inhibitor, 5-aza 2′-deoxycytidine. The expression levels of miR-193a expression also increased by 2.5 to 4.0 fold, upon treatment with 5-aza 2′-deoxycytidine [23] (Fig. 1h). Therefore, the lack of miR-193a expression in the Group 3 cells is likely to be due to promoter hypermethylation.

### MiR-193a inhibited proliferation, induced apoptosis, and increased radiation sensitivity of MYC overexpressing group 3 cell lines

**Fig. 2:**
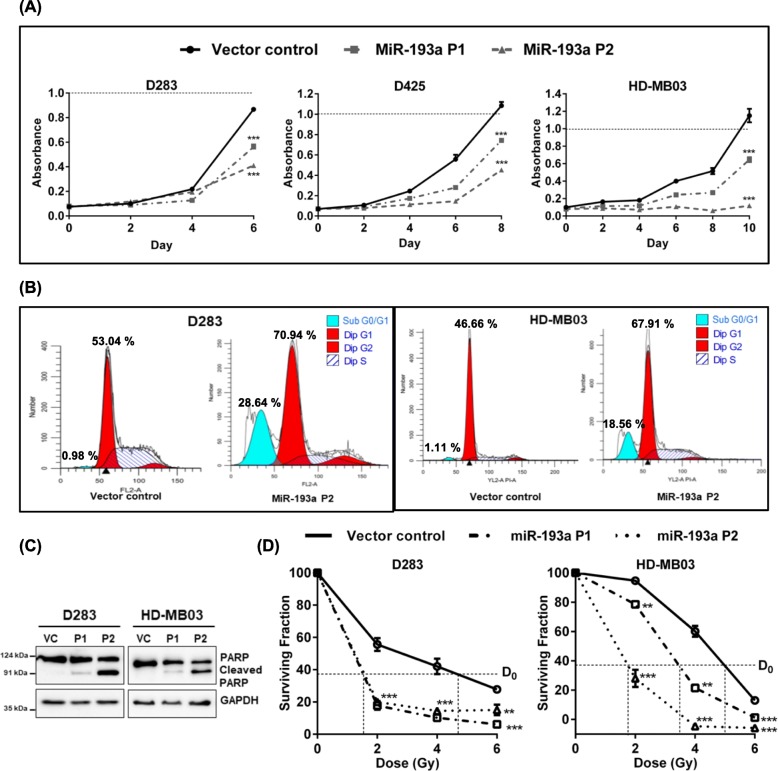
Effect of miR-193a expression on the proliferation and radiation sensitivity of the medulloblastoma cells evaluated by the MTT assay and the cell cycle analysis by the flow cytometry assay. Medulloblastoma cells expressing the parental pTRIPZ vector alone (Vector control) and the P1/P2 populations expressing doxycycline-inducible miR-193a-pTRIPZ construct were treated with doxycycline before evaluation by the MTT assay or the flow cytometry analysis. **a** Growth curves of the indicated medulloblastoma cells evaluated by the MTT assay. **b** Cell cycle analysis of indicated medulloblastoma cells, stained with Propidium iodide and, evaluated by flow cytometry. **c** Western blot analysis of PARP, a marker of apoptotic cell death, in the indicated medulloblastoma cells. The blot was also probed with anti-GAPDH antibody to serve as a loading control. **d** Y-axis denotes the surviving fraction of the indicated medulloblastoma cells upon irradiation at a dose ranging from 2 Gy to 6 Gy. VC: Vector control; **, *** and ns indicate *p* < 0.001, *p* < 0.0001, respectively

The recently established HD-MB03 cell line [12] and the D425 cell line overexpress MYC as a result of the amplification of the gene, while the D283 cell line overexpresses MYC without amplification [13]. None of these Group 3 cell lines express miR-193a (Supplementary Fig. 1A). The cell lines were transduced with the pTRIPZ lentiviral vector expressing miR-193a in a doxycycline-inducible manner. Stably transduced polyclonal populations (P1, P2) were selected in the presence of puromycin. The expression levels of miR-193a in the P1, P2 populations upon doxycycline induction were in the range of RQ 0.5 to 4.5 similar to those in the WNT subgroup tumors (Supplementary Fig. 2A, Supplementary Fig. 1A). MiR-193a expression was found to inhibit the growth of all the cell lines by 40 to 90% depending upon the level of exogenous miR-193a expression, as studied by the MTT assay (Fig. 2a). The cell cycle analysis by flow cytometry showed the arrest of 76% (± 3.3) of D283 cells, and 67.11% (± 2.74) of HD-MB03 cells in the G0/G1 phase of the cell cycle upon miR-193a expression as compared to 57.51% (± 2.25), and 45% (± 1.66) of the vector control cells, respectively (Fig. 2b, Supplementary Fig. 2B). The analysis showed 20 to 30% of miR-193a expressing cells in the sub-G0/G1 fraction as well, indicating the induction of cell death upon miR-193a expression. Cleavage of PARP [Poly (ADP-Ribose) Polymerase] upon miR-193a expression indicated apoptotic cell death (Fig. 2c). The MTT assay was used to study the effect of miR-193a expression upon the radiation sensitivity of medulloblastoma cells. Medulloblastoma vector control cells and the P1, P2 population cells were treated with doxycycline for 48 h and then irradiated using a telecobalt machine at a dose ranging from 2 Gy to 6 Gy. The cell growth was monitored by the MTT assay. MiR-193a expression brought about 2.34 to 3.3 fold and 1.7 to 2.8 fold increase in the radiation sensitivity of D283 and HD-MB03 cells, respectively as evaluated based on the reduction in the D_0_ dose of radiation, which is the dose at 37% surviving fraction of the cells (Fig. 2d).

### MiR-193a expression inhibited the anchorage-independent growth and tumorigenicity of medulloblastoma cells

**Fig. 3:**
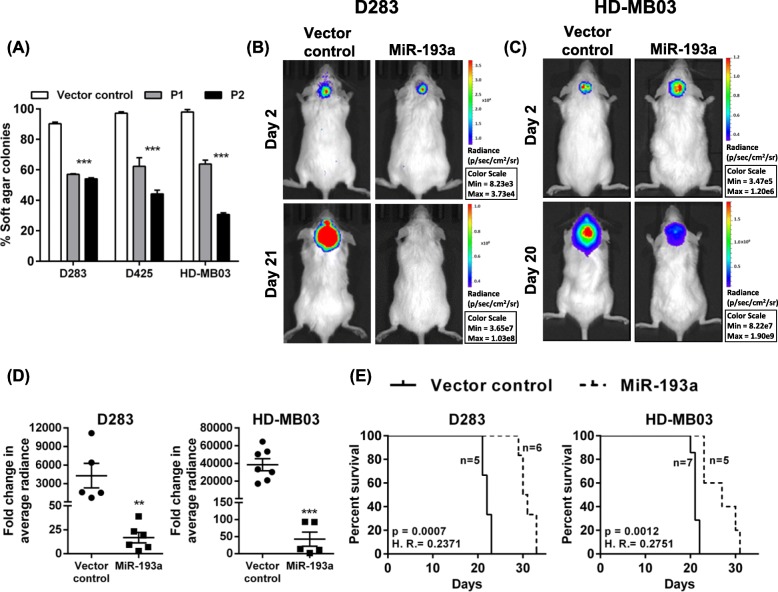
Effect of miR-193a expression on the anchorage-independent growth and the tumorigenicity of medulloblastoma cells. **a** Y-axis denotes the number of soft agar colonies formed by the indicated medulloblastoma cells, upon doxycycline treatment as a percentage of the untreated control cells. **b**, **c** In vivo bioluminescence images of the orthotopic tumors at day 2 and day 21 post-injection of doxycycline-treated vector control cells (Vector control) or miR-193a expressing medulloblastoma cells (MiR-193a) in the cerebellar region of the NOD/SCID mice. **d** Y-axis denotes the fold change in the tumor growth on day 21 compared to that on day two, as evaluated by the change in the average luminescence of the tumor area. **e** Kaplan Meier survival analysis of the mice injected with the doxycycline-treated vector control or miR-193a expressing medulloblastoma cells of the indicated cell line. P1, P2: Medulloblastoma cells expressing miR-193a upon doxycycline treatment. **, *** indicate *p* < 0.001, *p* < 0.0001 respectively. H.R. = Hazard Ratio

The soft agar colony formation assay was used for studying the effect of miR-193a expression upon the anchorage-independent growth of medulloblastoma cells. MiR-193a expression reduced the ability of medulloblastoma cells to form colonies in soft agar by 40 to 70% in all three cell lines (Fig. 3a, Supplementary Fig. 3A). The tumorigenic potential of miR-193a expressing D283 and HD-MB03 cells was studied by orthotopic injection of the cells in the cerebella of the immunodeficient NOD/SCID mice (Fig. 3b, c). The cells were engineered to express the firefly luciferase gene so that in vivo growth of the tumors could be monitored by the bioluminescence imaging. In vivo bioluminescence imaging showed a 200 to 800 fold decrease in the growth of tumors derived from the miR-193a expressing cells (Fig. 3d, Supplementary Fig. 3B). MiR-193a expression also increased the median survival of the tumor-bearing mice by about 30% as judged by the Kaplan Meier survival analysis (Fig. 3e).

### Transcriptome analysis showed downregulation of known and novel targets of miR-193a in medulloblastoma cells, upon miR-193a expression

**Fig. 4:**
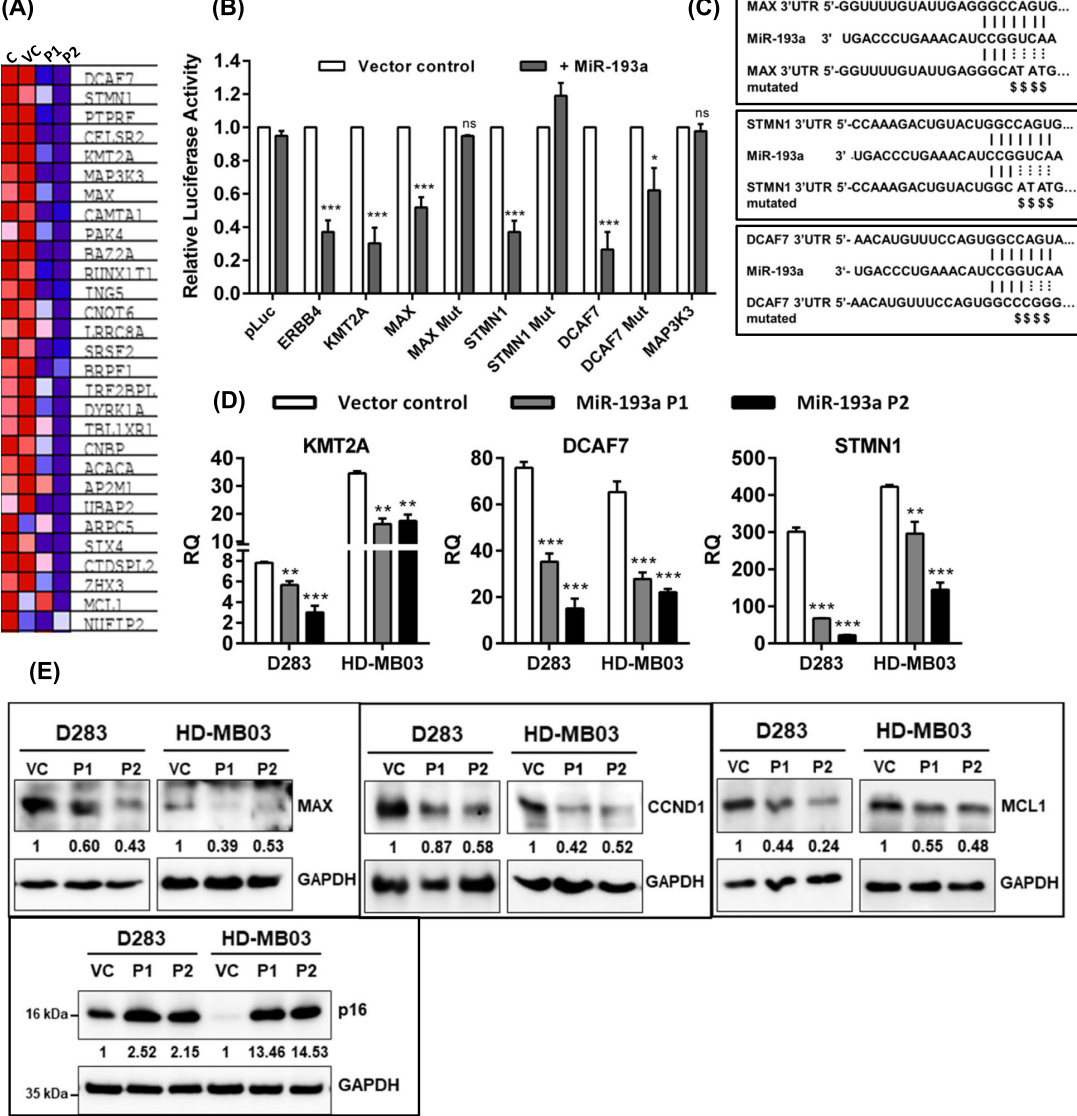
Identification of genes targeted by miR-193a by the transcriptome sequencing of the HD-MB03 medulloblastoma cells expressing miR-193a, validation of known and novel miR-193a targets. **a** GSEA analysis showed enrichment of miR-193a targets (Net Enrichment Score = 1.67; *p* = .003) in the genes differentially expressed upon the miR-193a expression as identified by the transcriptome sequencing. Heat map generated by this GSEA analysis shows downregulation of known and putative miR-193a targets in the P1, P2, the two independent cell populations expressing miR-193a as compared to the parental control [C] and the vector control [VC] cells. The expression values are represented as colors, where the range of colors (red, pink, light blue, dark blue) shows the range of expression values (high, moderate, low, lowest). **b** Y-axis denotes the relative activity of the luciferase reporter of the 3′-UTR construct of the indicated gene before and after miR-193a expression. Mut: Mutant 3′-UTR construct. **c** Mutations introduced in the miR-193a binding site by the site-directed mutagenesis in the 3′-UTR constructs of the indicated gene. **d** Real-time RT-PCR analysis of miR-193a target genes, upon doxycycline induction of miR-193a expression, in the P1 and P2 populations as compared to the doxycycline-treated vector control cells. **e** Western blot analysis of miR-193a targets MAX, CCND1, MCL1, and that of the cell cycle inhibitor p16 (CDKN2A) in the P1, P2 populations upon miR-193a expression as compared to the doxycycline-treated vector control cells. The expression levels of GAPDH, a housekeeping gene, were used as a loading control. The numbers below each blot indicate the fold change in the expression levels of the indicated gene in the P1, P2 population as compared to that in the vector control cells. *, **, *** and ns indicate *p* < 0.01, *p* < 0.001, *p* < 0.0001 and non-significant, respectively

Transcriptome sequencing was carried out to identify the genes differentially expressed upon miR-193a expression in order to delineate the molecular mechanism underlying the tumor-suppressive effect of miR-193a. Libraries prepared from the poly-A enriched RNA fraction of doxycycline-treated parental HD-MB03 cells, vector control cells, and the P1, P2 populations expressing miR-193a were subjected to high-throughput sequencing on the Illumina HiSeq 2500 platform. The genes significantly differentially expressed upon miR-193a expression were identified using the DESeq2 software. As many as 703 genes were differentially (p_adj_ < 0.05) expressed upon miR-193a expression (Supplementary Table 2). The gene set enrichment analysis (https://software.broadinstitute.org/gsea) showed 1.67 fold enrichment of miR-193a targets like *KMT2A*, *MCL1,* and several putative miR-193a targets like *DCAF7*, *STMN1*, *MAX*, and *MAP3K3* among the genes significantly downregulated upon miR-193a expression (Fig. 4a). The 3′-UTRs of the putative miR-193a target genes were cloned downstream of the luciferase cDNA in the reporter vector. The luciferase reporter activity of these 3′-UTR constructs was assayed upon transfection into the HEK293FT cells. The reporter activity decreased by 40 to 70% when the miR-193a expression construct was co-transfected in the case of known miR-193a targets *ERBB4*, *KMT2A,* and potential targets *MAX*, *STMN1*, and *DCAF7* but not in the case of *MAP3K3* (Fig. 4b). *MAX*, *DCAF7,* and *STMN1* were further validated to be direct targets of miR-193a by the site-directed mutagenesis of the miR-193a binding site in their 3′-UTRs. MiR-193a mediated reduction in the luciferase reporter activity was abrogated upon mutations in the miR-193a binding sites (Fig. 4b, c). The real-time RT-PCR analysis further validated the reduction in the expression levels of *KMT2A*, *DCAF7,* and *STMN1* in HD-MB03 cells and D283 cells upon miR-193a expression (Fig. 4d). MiR-193a mediated decrease in the expression levels of known targets CCND1, MCL1, and the novel target MAX in the medulloblastoma cells was validated by the western blot analysis (Fig. 4e). Furthermore, consistent with the growth arrest of the cells in the G0/G1 phase, the expression of p16, a CDK4/CDK6 inhibitor, was found to be upregulated in medulloblastoma cells upon miR-193a expression (Fig. 4e).

### MiR-193a expression brought about widespread repression of gene expression with the decrease in the global levels of histone marks of active chromatin

**Fig. 5:**
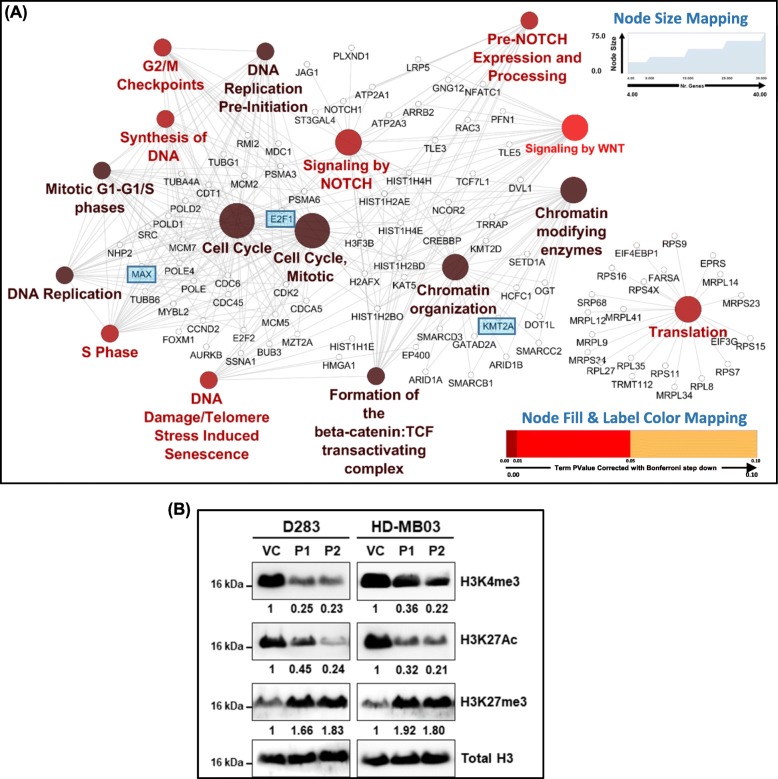
Interaction network analysis of the pathways significantly enriched in the genes downregulated upon miR-193a expression, and the change in the expression levels of the core histone marks in the medulloblastoma cells upon miR-193a expression. **a** The 439 genes significantly downregulated (padj < 0.05, log2 fold change < 0.8) upon miR-193a expression were analyzed for their interaction and functional enrichment (p_adj_ < 0.01) in the pathways from the KEGG and Reactome database using the ClueGO application in the Cytoscape software. MiR-193a targets E2F1, MAX, KMT2A are highlighted in the interaction network. The fill color of the node and that of the label indicate the statistical significance of the enrichment of the pathway. The higher size of the node indicates a higher number of interacting genes. **b**. Western blot analysis of the histone marks, H3K4me3, H3K27ac, and H3K27me3 in the medulloblastoma cells. The numbers below each band indicate the fold change in the expression levels of the histone mark in the P1, P2 populations expressing miR-193a as compared to the vector control cells after normalization using the total histone H3 levels as the loading controls

The protein-protein interaction network analysis of the genes significantly downregulated upon miR-193a expression in the HD-MB03 cells was carried out using the ClueGo, a Cytoscape plug-in software (http://apps.cytoscape.org/apps/cluego). The network was analyzed for the enrichment of biological pathways from the KEGG and the Reactome databases. The interaction network analysis of the 459 genes downregulated (log_2_fold change = 0.8; p_adj_ < 0.05; Supplementary Table 2) upon miR-193a expression showed significant enrichment (p_adj_ < 0.01) of several pathways including the cell cycle regulation, DNA replication/DNA synthesis, chromatin remodeling, NOTCH signaling, WNT signaling, and the translation machinery (Fig. 5a). The decrease in the expression levels of proliferation-related targets of miR-193a like *E2F1*, *CCND1,* and several other cell cycle regulators like *CDK2*, *CCND2* is consistent with the growth inhibition of medulloblastoma cells upon the expression of miR-193a. The genes encoding several components of the DNA replication machinery like DNA polymerase delta (*POLD1*, *POLD2*), DNA polymerase epsilon (*POLE*), and those encoding components of replisome like *CDC45*, *MCM2*, *MCM5*, *MCM7* were among the genes downregulated upon miR-193a expression in the medulloblastoma cells. Therefore, miR-193a appears to also inhibit DNA replication in the medulloblastoma cells. The downregulation of several NOTCH signaling regulators like *NOTCH1*, *JAG1*, *PLXND1,* and WNT pathway genes like *TCF7L1*, *LRP5*, *DVL1*, and *TLE3* suggests inhibition of WNT and NOTCH signaling pathways upon miR-193a expression.

MiR-193a expression also resulted in a reduction in the expression of several genes belonging to the chromatin organization. The genes belonging to the SWI/SNF remodeling complex, histone encoding genes, and histone-modifier genes were downregulated upon miR-193a expression. The SWI/SNF complex genes included *ARID1A*, *ARID1B*, *SMARCB1*, *SMARCD3*, and *SMARCC2*, while the histone encoding genes included *HIST1H4E*, *HIST1H2BD*, and *H3F3B.* The histone modifier genes included known miR-193a target *KMT2A*, as well as *KMT2D*, and *KAT5. KMT2A* encodes the catalytic subunit of the MLL1 multiprotein complex that is responsible for the H3K4 trimethylation [24]. The western blot analysis of the total levels of H3K4me3 (Histone H3 trimethyl K4) and H3K27ac (Histone H3 acetyl K27), which are marks of active gene expression decreased by 55 to 78% upon miR-193a expression in the medulloblastoma cells (Fig. 5b). On the other hand, the total levels of H3K27me3 (Histone H3 trimethyl K27), an epigenetic mark of repression of gene expression, increased by 1.66 to 2 fold upon miR-193a expression (Fig. 5b) indicating widespread repression of gene expression.

## Discussion

Among the four molecular subgroups of medulloblastomas, Group 3 has the worst five-year survival rate (< 50%), while the WNT subgroup has the best long term survival rate of over 90% [25, 26]. MiR-193a is almost exclusively expressed in the WNT subgroup medulloblastomas. In the present study, MYC was found to upregulate the activity of the miR-193a promoter and induce the expression of miR-193a. *MYC* is a crucial oncogenic target of the WNT signaling pathway. Therefore, miR-193a expression could be induced by MYC in the WNT subgroup medulloblastomas. Group 3 tumors also express MYC, and those that carry *MYC* amplification have the worst prognosis [26]. However, the majority of Group 3 tumors do not express miR-193a. The CpG island in the miR-193a promoter region has been reported to bring about silencing of miR-193a expression in several cancers like oral cancer [27], non-small cell lung cancer [28], hepatocellular carcinoma [16], and acute myeloid leukemia [29]. Several CpG residues in the miR-193a promoter region were found to be methylated in the non-WNT subgroup tumors in the MAGIC cohort of 763 medulloblastomas [4]. Furthermore, expression levels of miR-193a were restored in the Group 3 medulloblastoma cell lines upon treatment with the DNA methylation inhibitor, 5-aza-deoxycytidine. Thus, miR-193a expression appears to be induced by MYC in the WNT subgroup and repressed in the non-WNT subgroups as a result of promoter hypermethylation.

Restoration of miR-193a expression inhibited the growth and malignant potential of the Group 3 cell lines, which indicates that miR-193a acts as a tumor suppressor in the medulloblastoma pathogenesis. MiR-193a expression in the Group 3 cells resulted in a widespread change in their expression profile. *STMN1* encoding Stathmin 1 was identified as a novel miR-193a target. It is a microtubule destabilizing protein that plays a crucial role in the cell division. Stathmin 1 depletion is known to cause cell cycle arrest and apoptosis in various cancer cells [30, 31]. *DCAF7*, another novel miR-193a target, encodes a WD40 repeat-containing protein. DCAF7 is reported to promote myogenesis by phosphorylating the C-terminal domain of RNA polymerase II [32]. DCAF7 has also been found to be necessary for maintaining cellular levels of the ERCC1-XPF endonuclease complex involved in DNA repair [33]. The decrease in the levels of DCAF7 can lead to impairment of DNA repair, thereby increase the radiation sensitivity of medulloblastoma cells upon miR-193a expression. Thus, the downregulation of *STMN1* and *DCAF7* may contribute to the tumor-suppressive activity of miR-193a by decreasing proliferation, inducing apoptosis, and increasing radiation sensitivity of medulloblastoma cells.

The reduction in the expression levels of miR-193a targets like *E2F1*, *CCND1*, *MAX*, and *STMN1* is consistent with the downregulation of the WNT, MYC signaling genes, as well as, the cell cycle regulatory genes upon miR-193a expression. Several genes encoding minichromosome maintenance proteins like *MCM2*, *MCM5*, *MCM7* were downregulated upon miR-193a expression. High expression of MCM proteins has been reported to be important for cancer cells in order to survive replication stress [34]. Therefore, the reduction in the expression levels of MCM proteins in addition to MCL1, the anti-apoptotic target of miR-193a, could contribute to the induction of apoptosis. MCM proteins are also direct targets of checkpoint pathways and are required for the checkpoint execution in response to the radiation-induced DNA damage [35]. The expression levels of several genes encoding DNA polymerases decreased upon miR-193a expression. Apart from DNA replication, DNA polymerases like *POLD1*, *POLD2* also play a role in DNA repair [36]. The decrease in the levels of the MCM proteins and the DNA polymerases, therefore, could contribute to the increase in the radiation sensitivity of medulloblastoma cells upon miR-193a expression.

The large scale change in the expression profile in the medulloblastoma cells also includes several chromatin modifier genes indicating chromatin remodeling upon miR-193a expression. *KMT2A* encoding histone H3K4 methyltransferase is a recently reported target of miR-193a [37]. The reduction in the *KMT2A* levels is likely to contribute to the global reduction in the H3K4me3 levels in medulloblastoma cells, thereby repressing gene expression upon miR-193a expression. However, the widespread repression of gene expression is unlikely to be the result of downregulation of *KMT2A* alone.

MAX, the obligate heterodimerization partner of MYC, was identified as a novel target of miR-193a. MYC family transcription factors include *MYC*, *MYCN*, and *MYCL* oncogenes [38]. Their expression is upregulated in response to growth factors, mitogens, and cytokines. On the other hand, MXD/MGA/MNT family proteins are believed to antagonize the pro-proliferative activity of the MYC family proteins. Unlike MYC, MAX can form homodimers and heterodimers with MYCN and MYCL, as well as with MXD/MGA/MNT family proteins. In the present study, *MAX* was found to be a direct target of miR-193a, while MYC was found to induce miR-193a expression. The downregulation of *MAX* by miR-193a could suppress the activity of MYC since it is an obligate hetero-dimerization partner of MYC. *MAX* deletion has been reported to destabilize MYC and abrogate *MYC* driven lymphomagenesis [39]. MiR-193a induced by MYC, therefore, seems to act as a feedback inhibitor of MYC signaling. MAX, in turn, has been reported to suppress miR-193a expression [40]. Thus, miR-193a and MAX appear to negatively regulate each other’s expression, thereby modulating the activity of the MYC transcription factor family. In cancer cells having high MYC expression, MYC accumulates in the promoter regions of active genes and brings about their transcriptional amplification [41]. Therefore, miR-193a mediated decrease in the activity of MYC is likely to contribute to the miR-193a mediated widespread repression of gene expression in the MYC overexpressing Group 3 medulloblastoma cells.

Among the four molecular subgroups of medulloblastomas, Group 3 tumors carrying *MYC* /*MYCN* amplification are the most aggressive tumors with the worst prognosis. MYC regulates a wide variety of cellular functions, including cell cycle, protein synthesis, metabolism, and stem cell self-renewal. MYC is a highly challenging target. It is a transcription factor that lacks a specific active site for designing small molecular inhibitors. Therefore, small molecular inhibitors have been designed to target its interaction with MAX, its obligate heterodimerization partner [42]. These inhibitors, however, are challenging to deliver to the nuclear compartment. MiR-193a not only targeted *MAX* but several proliferation-related genes like *CCND1*, *E2F1*, *STMN1*, and chromatin modifier gene *KMT2A* that brought about widespread repression of gene expression in the *MYC* amplified Group 3 medulloblastoma cells. Thus, miR-193a countered the transcription amplifying effect of MYC resulting in the inhibition of growth and tumorigenic potential of the *MYC* amplified medulloblastoma cells. Furthermore, miR-193a also brought about a substantial increase in the radiation sensitivity of the medulloblastoma cells, which is likely to be the result of the downregulation of DNA replication, DNA repair machinery, and decrease in the levels of its anti-apoptotic target MCL1. MiR-193a, therefore, has therapeutic potential in the treatment of this highly aggressive cancer. *MYC* oncogene is deregulated in over 50% of human cancers [43, 44]. MiR-193a could be effective for the treatment of not only Group 3 medulloblastomas but possibly other MYC overexpressing aggressive cancers as well.

Cancer is the result of multiple genetic alterations. MicroRNAs, which are natural inhibitors of multiple genes, are attractive therapeutic molecules provided they are exclusively tumor-suppressive or oncogenic. MiR-193a has been reported to be tumor-suppressive in multiple cancers by downregulating several oncogenic targets [45–48]. Hence it is potentially a good therapeutic small molecule. Although microRNA based drugs have not yet entered the clinics, the safety of microRNA based therapeutics has been demonstrated in several phase I clinical trials [49, 50]. Rapid advances are being made in designing and optimizing various approaches for targeted microRNA delivery that include liposomes, nanoparticles, exosomes, as well as viral vectors like adeno-associated vectors [51, 52]. In the case of brain tumors, the blood-brain barrier also needs to be overcome. Non-invasive focused ultrasound treatment that disrupts the blood-brain barrier in a targeted area is one of the potential approaches for targeted delivery into brain tumors [53]. Efficacy and safety of miR-193a need to be evaluated in pre-clinical models using patient-derived xenografts for utilizing the therapeutic potential of this tumor-suppressive microRNA.

## Conclusions

MiR-193a, a WNT subgroup-specific microRNA, is downregulated as a result of hypermethylation of the CpG island in its promoter region in the three non-WNT subgroups. The expression of miR-193a inhibited growth, tumorigenicity, and increased radiation sensitivity of MYC amplified/overexpressing Group 3 medulloblastoma cells. *MAX*, *DCAF7*, and *STMN1* were identified as novel targets of miR-193a that could contribute to its tumor-suppressive effect. MiR-193a is induced by MYC and targets *MAX*, its obligate heterodimerization partner, and thus appears to act as a feedback inhibitor of the MYC signaling. The expression of miR-193a in the medulloblastoma cells brought about widespread repression of gene expression that included genes involved in the WNT signaling, NOTCH signaling, cell cycle regulators, DNA replication as well as chromatin organization and modification. This widespread repression of gene expression was found to be accompanied by a substantial decrease in the global levels of H3K4me3, H3K27ac, the histone marks of active chromatin, and an increase in H3K27me3, a mark of repressed chromatin. In cancer cells having high MYC expression, MYC accumulates in the promoter regions of active genes and brings about their transcriptional amplification [41]. MiR-193a expression, on the other hand, led to global repression of gene expression accompanied by the tumor-suppressive effect in MYC overexpressing Group 3 cells. MiR-193a, therefore, has therapeutic potential for the treatment of not only the Group 3 medulloblastomas but possibly other MYC overexpressing aggressive cancers as well.

## Supplementary information

**Additional file 1:Figure S1**. MiR-193a and MYC expression levels in the Indian cohort and the methylation status of the CpG island in the miR-193a promoter region in the MAGIC cohort and the medulloblastoma cell lines. MiR-193a (A) and *MYC* (B) expression levels in the four molecular subgroups of 103 medulloblastomas from the Indian cohort [10] and the Group 3 medulloblastoma cell lines D283, D425, HD-MB03, and Daoy. (C) Methylation status of the indicated CpG probe in the miR-193a promoter CpG island in the four molecular subgroups of medulloblastoma from the MAGIC cohort [4]. (D) Analysis of the miR-193a promoter CpG island methylation status using the methylation-specific PCR in the medulloblastoma cell lines using two distinct sets of primers (I, II). The presence of PCR amplified product of the expected size in the PCR reaction using the primers specific for methylated or un-methylated CpG residues indicate methylation or unmethylated CpG residues, respectively in the indicated cell line or in the normal cerebellum.

**Additional file 2:Figure S2**. Expression levels of miR-193a in medulloblastoma cell lines after exogenous expression and their effect on cell growth studied by flow cytometry analysis. (A) MiR-193a expression levels in the parental D283, D425, and HD-MB03 cells and their P1 or P2 polyclonal populations expressing pTRIPZ-miR-193a construct, and the vector control cells expressing pTRIPZ vector alone or control, the parental cells before and after treatment with doxycycline for 48 h. (B) Y-axis denotes the percentage of cells of the indicated cell line in various phases of the cell cycle as evaluated by the flow cytometry analysis.

**Additional file 3:Figure S3**. Effect of miR-193a expression on the anchorage-independent growth and tumorigenicity of medulloblastoma cells. A. Y-axis denotes the number of soft agar colonies formed by the medulloblastoma cells, before and after doxycycline treatment. B. Y-axis denotes the average radiance of the orthotopic tumors of the vector control or miR-193a expressing populations of D283, HD-MB03 cells at the indicated time points *** indicates *p* < 0.001.

**Additional file 4:Table S1**. The list and the DNA sequences of the primers used in the study.

**Additional file 5:Table S2**. The genes significantly downregulated upon miR-193a expression (p_adj_ < 0.05) in the HD-MB03 cells identified by the DESeq2 analysis of the transcriptome data. The genes are listed in the decreasing order of log_2_ fold change.

## Data Availability

The datasets used during the current study are available from the corresponding author on reasonable request.

## Abbreviations

PCR: Polymerase Chain Reaction
bp: base pair
CMV: Cytomegalovirus
CI: Confidence Interval
GSEA: Gene Set Enrichment Analysis
TSS: Transcription Start Site
H3K4me3: Trimethylated Histone H3 protein on 4th Lysine residue
H3K27me3: Trimethylated Histone H3 protein on 27th Lysine residue
H3K27ac: Histone H3 protein acetylated on 27th Lysine residue
