# Supplementary figures and images for: Restoration of miR-193a expression is tumor-suppressive in MYC amplified Group 3 medulloblastoma
Source: Acta Neuropathol Commun. 2020 May 14;8:70. doi: 10.1186/s40478-020-00942-5 (PMC7227220; doi:10.1186/s40478-020-00942-5)

## Slide 1
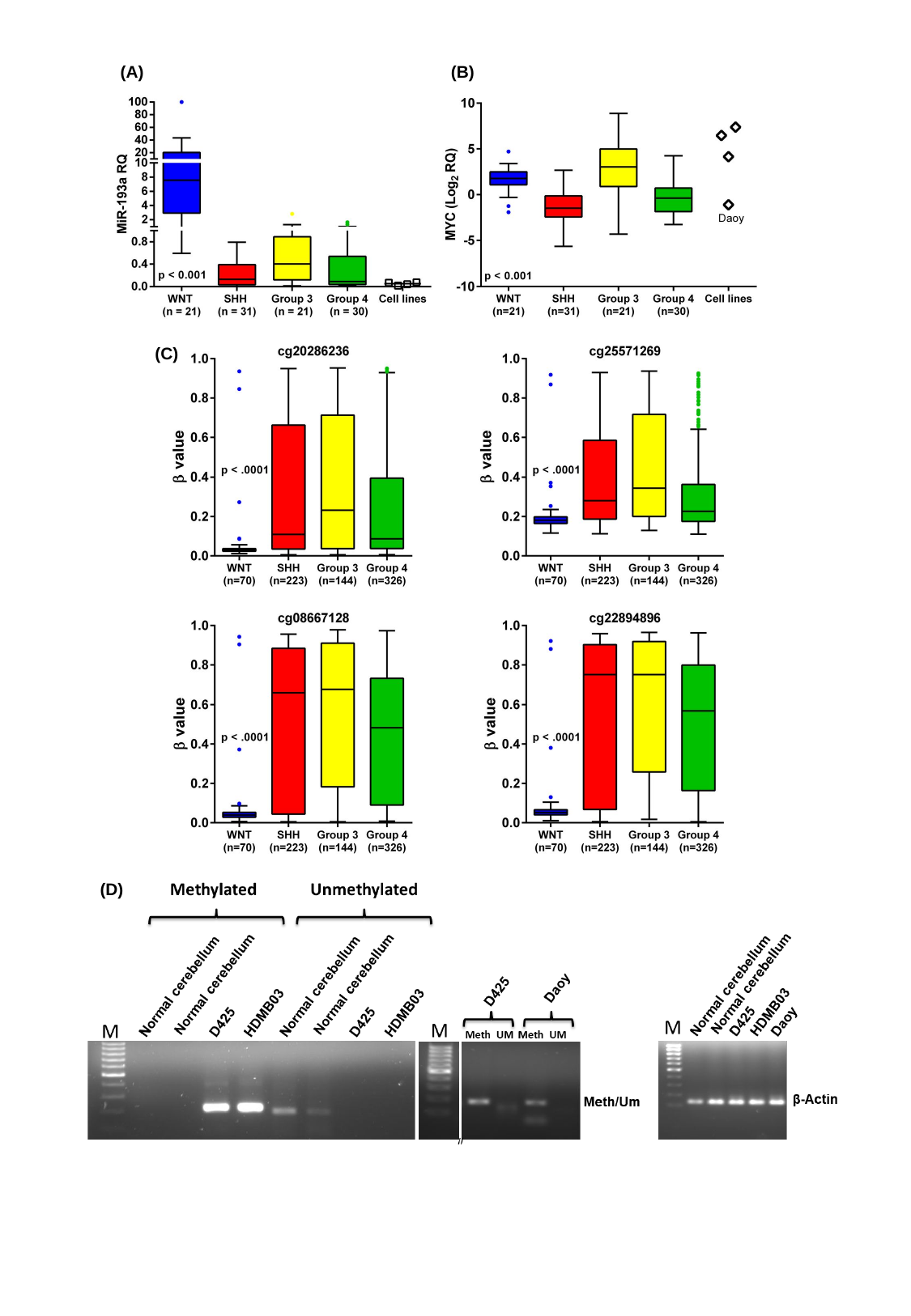

(A)
(B)
(C)
(D)

Supplement: Supplementary file 1 — Additional file 1:Figure S1. MiR-193a and MYC expression levels in the Indian cohort and the methylation status of the CpG island in the miR-193a promoter region in the MAGIC cohort and the medulloblastoma cell lines. MiR-193a (A) and MYC (B) expression levels in the four molecular subgroups of 103 medulloblastomas from the Indian cohort [10] and the Group 3 medulloblastoma cell lines D283, D425, HD-MB03, and Daoy. (C) Methylation status of the indicated CpG probe in the miR-193a promoter CpG island in the four molecular subgroups of medulloblastoma from the MAGIC cohort [4]. (D) Analysis of the miR-193a promoter CpG island methylation status using the methylation-specific PCR in the medulloblastoma cell lines using two distinct sets of primers (I, II). The presence of PCR amplified product of the expected size in the PCR reaction using the primers specific for methylated or un-methylated CpG residues indicate methylation or unmethylated CpG residues, respectively in the indicated cell line or in the normal cerebellum. [file 40478_2020_942_MOESM1_ESM.pptx]

## Slide 1
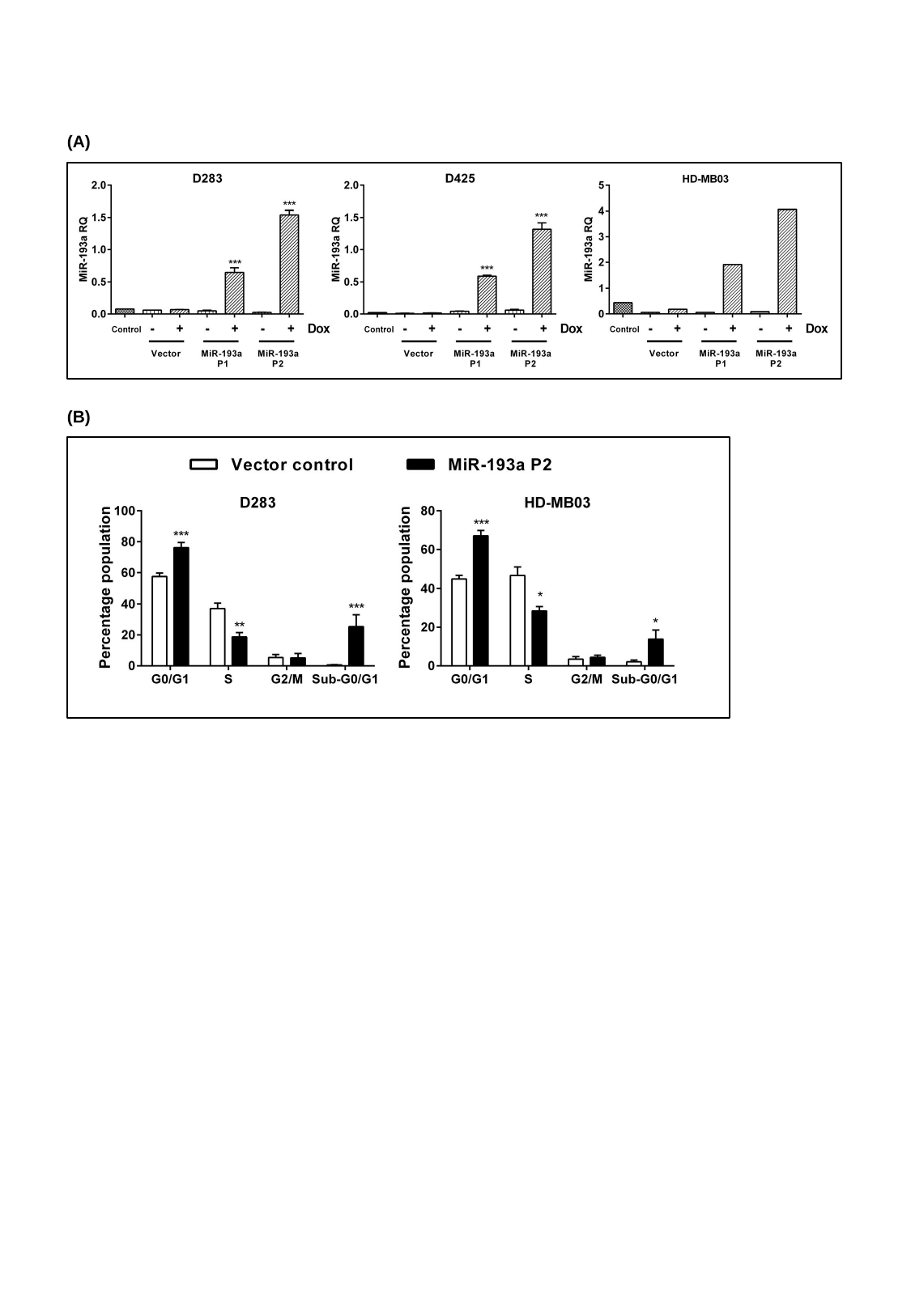

(A)
(B)

Supplement: Supplementary file 2 — Additional file 2:Figure S2. Expression levels of miR-193a in medulloblastoma cell lines after exogenous expression and their effect on cell growth studied by flow cytometry analysis. (A) MiR-193a expression levels in the parental D283, D425, and HD-MB03 cells and their P1 or P2 polyclonal populations expressing pTRIPZ-miR-193a construct, and the vector control cells expressing pTRIPZ vector alone or control, the parental cells before and after treatment with doxycycline for 48 h. (B) Y-axis denotes the percentage of cells of the indicated cell line in various phases of the cell cycle as evaluated by the flow cytometry analysis. [file 40478_2020_942_MOESM2_ESM.pptx]

## Slide 1
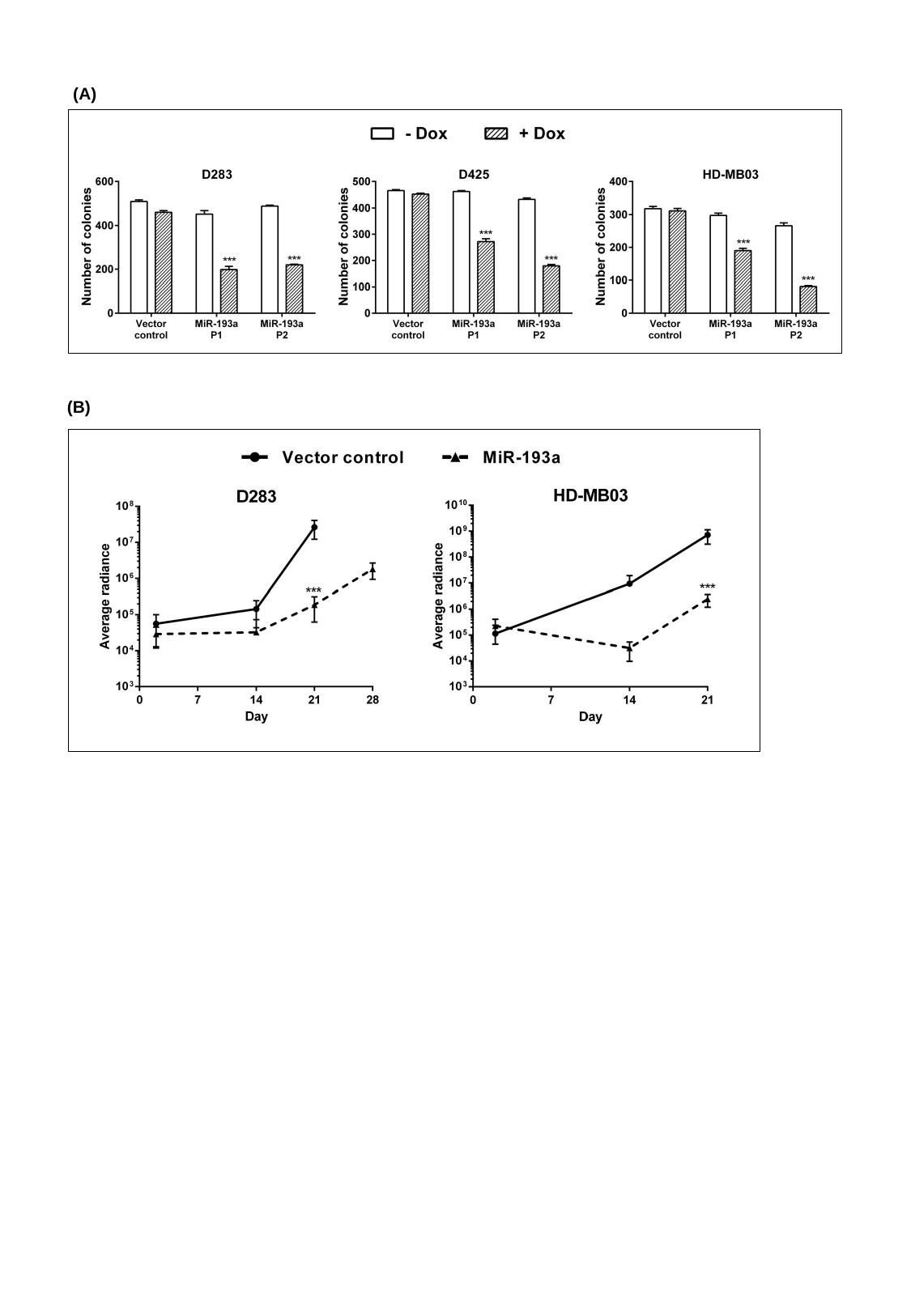

(A)
(B)

Supplement: Supplementary file 3 — Additional file 3:Figure S3. Effect of miR-193a expression on the anchorage-independent growth and tumorigenicity of medulloblastoma cells. A. Y-axis denotes the number of soft agar colonies formed by the medulloblastoma cells, before and after doxycycline treatment. B. Y-axis denotes the average radiance of the orthotopic tumors of the vector control or miR-193a expressing populations of D283, HD-MB03 cells at the indicated time points *** indicates p < 0.001. [file 40478_2020_942_MOESM3_ESM.pptx]
