# Supplementary material for: Restoration of miR-193a expression is tumor-suppressive in MYC amplified Group 3 medulloblastoma
Source: Acta Neuropathol Commun. 2020 May 14;8:70. doi: 10.1186/s40478-020-00942-5 (PMC7227220; doi:10.1186/s40478-020-00942-5)
Supplement: Supplementary file 4 — Additional file 4:Table S1. The list and the DNA sequences of the primers used in the study. [file 40478_2020_942_MOESM4_ESM.pdf]

Supplementary Table 1

The list and DNA sequence of the primers used

Primer name	Sequence (5'to 3')	Description
MiR-193a_Gen_F	GAGGAATTCGAGCGTCGTGTAACCCTTGG	Primers for amplification of the genomic region encoding miR-193a
MiR-193a_Gen_R	GGTATCTAGAGTCCCGTCTGTCCACTCAAC	
MiR-193a_Prom_F	TATGGTACCCGGTACTATGCTTGGCACT	Primers used for the amplification of upstream region of pri-miR-193a (-1292 to -158) along with restriction sites for cloning in pGL3 Basic vector.
MiR-193a_Prom_R	CCAGATATCCAAGGGTTACACGACGCTC	
MiR-193a_Prom_Mut1_F	GATGTGTCACCAGCGGGTGGAGCCAGGTCGG	Primers used for mutating E-box sequence in the upstream region of pri-miR-193a by site directed mutagenesis
MiR-193a_Prom_Mut1_R	CCGACCTGGCTCCACCCGCTGGTGACACATC	
MiR-193a Prom_Mut2_F	GTGTCACCAGCACGGGGAGCCAGGTCGGGC	
MiR-193a Prom Mut2_R	GCCCGACCTGGCTCCCCGTGCTGGTGACAC	
MiR-193a_MSP_Meth_F	GGGGACGTATTTCGAATTTC	Primers used for Methylation specific PCR for amplification of the CpG island in the miR-193a promoter region from bisulphite converted genomic DNA of medulloblastoma cell lines
MiR-193a_MSP_Meth_R	TAAAAAACAACCTAACCGAAACG	
MiR-193a_MSP_UnMeth_F	GGGGATGTATTTTGAATTTTGA	
MiR-193a_MSP_UnMeth_R	ACACACACCAACCCAAAAA	
Meth_ACTB_F1	TATATAGGTTGGGGAAGTTTG	Primers used for the amplification of *ACTB* gene from bisulphite converted genomic DNA of medulloblastoma cell lines
Meth_ACTB_R1	TATAAAAACATAAAACCTATAACC	
Meth ACTB_F2	TGGTGATGGAGGAGGTTTAGTAAGT	

Meth ACTB_R2	AACCAATAAAACCTACTCCTCCCTTAA	
**Primer name**	**Sequence (5'-3')**	**Description**
GAPDH _RT-PCR_F	GAAGGTCGGAGTCAACGGATT	Primers used for the Real Time RT_PCR analysis of the indicated genes
GAPDH_RT-PCR_R	GAGTTAAAAGCAGCCCTGGTG	
MYC_RT-PCR_F	GTAGTGGAAAACCAGCAGCC	
MYC_RT-PCR_R	CGAGTCGTAGTCGAGGTCAT	
WIF1_RT-PCR_F	GGAGACCTCTGTTCAAAGCCT	
WIF1_RT-PCR_R	ATTTGTTGGGTTCATGGCAGG	
DCAF7_RT-PCR_F	CCCATACCCCACCACAA	
DCAF7_RT-PCR_R	TTCACCAACCCTCCACAC	
KMT2A_RT-PCR_F	GCGGAGAGGATGAGCAAT	
KMT2A_RT-PCR_R	TTTCGGTCAGAGCCACTTC	
STMN1_RT-PCR_F	ATTCTCAGCCCTCGGTCA	
STMN1_RT-PCR_R	ACTTGCGTCTTTCTTCTGC	
ERBB4 _3'UTR_Gen_F	GTGGATCCGCCTCCACCTTACAGAC	Primers used for the amplification of 3'UTR sequences of the indicated genes along with restriction sites for cloning in pcDNA3.0-Luciferase (pLuc) 3'UTR reporter vector
ERBB4_3'UTR_Gen_R	TCTCTCGAGCCCTTCTCCTGCTCTACC	
KMT2A_3'UTR_Gen_F	ATAGGATCCATGGCAGAGACTTCCTTGT	
KMT2A_3'UTR_Gen_R	ATACTCGAGCAATGACCCGCTTTTCCTT	
MAX_ 3'UTR_Gen_F	CTATCTAGACCCAAAGCAGGAAGAAG	
MAX_3'UTR_Gen_R	TAATCTAGAGCAAATGCCAGGAACGG	
DCAF7_3'UTR_Gen_F	ATAGGATCCGCAGGGGCTTTTGTATTTCC	
DCAF7_3'UTR_Gen_R	ATACTCGAGCAGTGGTGCTTCAGGGTAA	
STMN1_3'UTR_Gen_F	ATAGGATCCTTTCTCCCCATCCCCTTC	

STMN1_3’UTR_Gen_R	ATACTCGAGCGTGCGGTCATTTGTGCGTT	
MAP3K3_3’UTR_Gen_F	TAAGGATCCTGTTTTTCCTTCCAATGTCTG	
MAP3K3_3’UTR_Gen_R	TAACTCGAGTAATGCGCAACACTGG	
MAX_3’UTR Gen_Mut_F	AATTCTTTGGGTGGCATAGAGGTTTTGTAT TGAGGATATCTGATGATGTT	Primers used for mutating miR-193a binding sequence in the 3’UTR region of indicated genes by site directed mutagenesis
MAX_3’UTR Gen_ Mut_R	GAAACCCACCGTATCTCCAAAACATAACTC CTATAGACTACTACAAAGCT	
DCAF7_ 3’UTR_Gen_Mut1_F	GAAACATGTTTCCAGTGGCCCGGGTGTCTT TCATTGCTTTGC	
DCAF7_3’UTR_Gen_ Mut1_R	GCAAAGCAATGAAAGACACCCGGGCCACT GGAAACATGTTTC	
STMN1_3’UTR_Gen_ Mut_F	ATATCCAAAGACTGTACTGGCATATGTCAT TTTATTTTTTCCC	
STMN1_3’UTR_Gen_ Mut_R	GGGAAAAAATAAAATGACATATGCCAGTA CAGTCTTTGGATAT	
