# Supplementary material for: Restoration of miR-193a expression is tumor-suppressive in MYC amplified Group 3 medulloblastoma
Source: Acta Neuropathol Commun. 2020 May 14;8:70. doi: 10.1186/s40478-020-00942-5 (PMC7227220; doi:10.1186/s40478-020-00942-5)
Supplement: Supplementary file 5 — Additional file 5:Table S2. The genes significantly downregulated upon miR-193a expression (padj < 0.05) in the HD-MB03 cells identified by the DESeq2 analysis of the transcriptome data. The genes are listed in the decreasing order of log2 fold change. [file 40478_2020_942_MOESM5_ESM.pdf]

Sr No	Gene Name	log2Fold Change	padj
1	MIR663A	-12.31	9.56E-05
2	MIR3648	-9.95	6.03E-05
3	RPPH1	-7.87	2.62E-07
4	RP11-372E1.1	-5.71	3.20E-16
5	HIST1H4E	-5.28	1.16E-23
6	HIST1H2BO	-5.23	2.54E-14
7	RN7SL2	-5.18	0.001157
8	RN7SL4P	-4.98	9.82E-11
9	HIST1H1E	-4.83	4.82E-14
10	RN7SL3	-4.74	1.15E-10
11	HIST1H2AE	-4.61	3.21E-12
12	AL161626.1	-4.22	1.67E-32
13	RN7SL1	-3.69	0.000486
14	CTD-2328D6.1	-3.66	2.21E-06
15	RP4-706A16.3	-3.59	1.07E-38
16	AC079949.1	-3.51	0.002335
17	FAUP1	-3.36	3.06E-13
18	CTB-36O1.7	-3.33	5.40E-15
19	RMRP	-3.24	1.28E-07
20	HMGN2P5	-3.05	4.17E-08
21	CYP51A1	-2.91	6.47E-07
22	PSMA6	-2.84	7.13E-07
23	MTRNR2L1	-2.79	1.15E-05
24	FOXO6	-2.73	1.50E-10
25	NPW	-2.65	1.50E-10
26	RP11-500C11.3	-2.65	3.17E-05
27	HIST1H4H	-2.55	4.53E-06
28	RN7SK	-2.30	1.51E-07
29	H1FX	-2.30	1.33E-16
30	RPL6P27	-2.22	1.38E-16
31	SGCA	-2.21	0.003036
32	MTATP8P1	-2.17	9.92E-14
33	JUND	-2.15	2.55E-09
34	SEMA6B	-2.08	6.73E-12
35	UNCX	-2.06	0.000268
36	GRIK3	-2.03	2.03E-14
37	UBE2S	-2.02	1.55E-13
38	CCDC85B	-2.01	8.05E-09
39	HSPB1	-1.98	1.51E-07
40	MRPL3P1	-1.98	6.93E-05
41	C12orf57	-1.97	2.62E-06
42	TPI1P1	-1.95	6.03E-06
43	POMK	-1.93	0.000738
44	FTH1	-1.89	9.58E-06

Sr No	Gene Name	log2Fold Change	padj
45	DCAF7	-1.87	1.80E-12
46	LMO1	-1.86	0.002627
47	HNRNPCP2	-1.82	1.45E-05
48	PPP1R14A	-1.79	4.33E-08
49	MYOD1	-1.78	1.15E-05
50	PWWP2B	-1.77	2.35E-05
51	CKB	-1.77	1.16E-07
52	CUX2	-1.76	2.31E-06
53	FAM222A	-1.75	0.000115
54	MTRNR2L12	-1.74	4.08E-07
55	ZHX2	-1.73	0.001794
56	RNF126	-1.73	1.98E-06
57	SMARCD3	-1.73	0.019189
58	STMN1	-1.71	1.20E-05
59	RGL1	-1.71	0.013529
60	HIVEP3	-1.71	0.00058
61	MEX3D	-1.68	0.001327
62	PLXNA2	-1.68	0.000209
63	NEB	-1.67	0.008979
64	RBM8B	-1.66	0.010129
65	GADD45G	-1.66	0.000959
66	NR2F6	-1.65	0.000337
67	WSCD1	-1.64	1.97E-06
68	RP11-766F14.2	-1.64	0.000311
69	C8orf82	-1.63	0.004918
70	FAM96B	-1.61	1.42E-05
71	TOMM5	-1.60	0.020285
72	IDH2	-1.60	1.79E-06
73	SLC44A3	-1.59	0.011267
74	RP11-20O24.4	-1.59	9.20E-09
75	KCNC1	-1.59	0.015796
76	DPP6	-1.59	0.010325
77	HIST1H2BD	-1.59	0.005437
78	IGSF9B	-1.57	0.002412
79	SEMA4B	-1.57	3.81E-05
80	MALAT1	-1.57	8.92E-05
81	H3F3C	-1.57	0.003253
82	H2AFX	-1.56	1.28E-06
83	RPL24P4	-1.56	4.10E-07
84	MRPL12	-1.54	0.008167
85	IL32	-1.53	0.038685
86	MXRA5P1	-1.53	0.001181
87	NFATC1	-1.53	0.028187
88	KCNH3	-1.52	0.003528

Sr No	Gene Name	log2Fold Change	padj
89	CHAF1B	-1.52	7.24E-05
90	ANO2	-1.52	0.005943
91	SHF	-1.52	8.75E-05
92	LSM6	-1.51	0.000144
93	BCORL1	-1.51	0.002795
94	EFS	-1.51	0.00823
95	CBFA2T3	-1.50	0.013037
96	LRFN1	-1.50	6.23E-05
97	SNHG16	-1.50	2.23E-05
98	KHDRBS3	-1.49	0.014688
99	NDRG1	-1.48	1.42E-05
100	AC007969.5	-1.47	0.00264
101	SCAND1	-1.47	0.000529
102	NGRN	-1.45	0.001431
103	NOL9	-1.45	0.000724
104	SIX2	-1.45	0.000138
105	PRELID1	-1.45	0.001625
106	PITX1	-1.44	9.92E-05
107	ORAI1	-1.44	0.007612
108	NDUFB2	-1.44	0.001053
109	KIF26A	-1.43	0.000936
110	TFAP4	-1.42	4.16E-05
111	SHMT2	-1.41	0.000225
112	ASS1	-1.40	2.51E-06
113	IGSF9	-1.40	0.000466
114	TP73	-1.40	0.001891
115	RPL8	-1.39	2.57E-08
116	FAM132B	-1.39	0.004837
117	NF2	-1.39	0.003457
118	TRMT112	-1.38	3.25E-05
119	RPL13P12	-1.38	0.000316
120	PKN3	-1.37	0.000449
121	CAMSAP3	-1.37	0.008669
122	SCARF2	-1.37	0.001929
123	UBALD1	-1.36	0.005908
124	SCNM1	-1.36	0.00058
125	PRKCD	-1.36	0.0301
126	C19orf43	-1.36	4.42E-06
127	FJX1	-1.36	0.000468
128	ARHGDIA	-1.35	2.51E-06
129	FGD6	-1.35	0.019368
130	NFKBIA	-1.35	0.001314
131	ELFN1	-1.34	1.66E-05
132	B4GALNT4	-1.34	2.01E-05

Sr No	Gene Name	log2Fold Change	padj
133	RPS9	-1.34	2.74E-07
134	CDCA7L	-1.33	6.02E-05
135	CISD3	-1.33	0.002645
136	MSANTD3	-1.33	0.030851
137	SLC29A1	-1.32	1.15E-05
138	IMPA2	-1.32	0.005185
139	ARID1A	-1.32	3.78E-06
140	JMJD8	-1.31	0.041265
141	PCBP1	-1.31	4.51E-06
142	CCDC23	-1.31	0.03417
143	ATP5J2	-1.31	0.001227
144	RXRG	-1.30	0.000664
145	FBRSL1	-1.30	0.000113
146	CXXC5	-1.30	0.004544
147	RPS6KA1	-1.30	0.042687
148	PLXND1	-1.30	0.000281
149	EYA2	-1.29	6.96E-05
150	MTND6P4	-1.29	3.95E-05
151	GABRQ	-1.29	0.000297
152	INHBE	-1.29	0.000504
153	FBXL16	-1.28	0.04466
154	PTPRF	-1.27	1.76E-06
155	NOTCH1	-1.27	0.000161
156	RBFA	-1.27	0.016915
157	DLX3	-1.27	0.020386
158	BCAT2	-1.26	0.004175
159	SYNDIG1	-1.26	0.000309
160	TMUB1	-1.26	0.020386
161	RMI2	-1.26	0.043756
162	NHSL1	-1.25	0.005043
163	EIF4EBP1	-1.25	0.004296
164	FOXRED2	-1.24	0.014117
165	SMAD6	-1.24	0.014405
166	DRAP1	-1.24	0.002511
167	RYR1	-1.24	0.045474
168	NCOR2	-1.23	1.15E-05
169	CLASRP	-1.23	0.000534
170	MESDC1	-1.23	0.003385
171	DYNLRB1	-1.22	0.000147
172	CECR2	-1.22	0.008906
173	MRPL41	-1.22	0.0146
174	TUBG1	-1.22	0.000453
175	SOCS7	-1.22	0.026753
176	CYC1	-1.22	0.000269

Sr No	Gene Name	log2Fold Change	padj
177	CHERP	-1.22	0.000848
178	PPIH	-1.21	0.020232
179	TIMM50	-1.21	6.96E-05
180	RHBDL3	-1.21	0.048187
181	DLL3	-1.21	0.004493
182	ZNF787	-1.21	0.007235
183	CLPP	-1.20	0.008866
184	LRP4	-1.20	0.001017
185	TLX2	-1.20	0.003869
186	CDC45	-1.20	0.002503
187	PNPLA2	-1.19	0.029024
188	COA6	-1.19	0.002735
189	GABBR2	-1.19	0.020232
190	HPCAL1	-1.19	0.002653
191	ATP5H	-1.19	0.000427
192	NELFA	-1.18	0.001445
193	KIF19	-1.18	0.002095
194	TLE3	-1.18	0.004903
195	RAC3	-1.18	0.000774
196	PFN1	-1.17	0.000216
197	HSPG2	-1.17	0.000315
198	CAPN15	-1.17	0.014571
199	MZT2A	-1.17	0.000522
200	TMEM258	-1.16	0.016703
201	ADCY9	-1.16	0.024639
202	NHP2	-1.15	0.003069
203	NME2P1	-1.15	0.021294
204	LBH	-1.15	0.002653
205	ZNF395	-1.14	0.005205
206	YIF1B	-1.14	0.018096
207	E2F2	-1.14	0.005534
208	NXT1	-1.14	0.035146
209	ATP2A1	-1.13	0.00451
210	ZFHX3	-1.13	0.009174
211	NTN3	-1.13	0.016746
212	SF3A2	-1.13	0.005907
213	MAP3K3	-1.13	0.002735
214	TUBB6	-1.13	0.000353
215	LAMA5	-1.13	0.000108
216	PIM3	-1.13	0.001361
217	HELLS	-1.13	0.003091
218	RP11-169K16.7	-1.13	0.022651
219	KATNB1	-1.12	0.000874
220	PQLC1	-1.12	0.034521

Sr No	Gene Name	log2Fold Change	padj
221	CCND2	-1.12	0.007751
222	TCEA3	-1.12	0.035146
223	FOXK1	-1.12	0.006726
224	CCNK	-1.12	0.002462
225	KMT2A	-1.12	0.002462
226	JAG1	-1.11	0.019189
227	ATP2A3	-1.11	0.007508
228	IRF2BP1	-1.11	0.030202
229	CHST14	-1.11	0.017882
230	BIN1	-1.10	0.000665
231	ZFAT	-1.10	0.00032
232	ARID3A	-1.10	0.035146
233	CLSTN1	-1.10	0.000522
234	DOT1L	-1.10	0.000315
235	POLR3K	-1.10	0.046007
236	CELSR2	-1.10	0.000664
237	RUSC1	-1.10	0.046309
238	HES6	-1.09	2.86E-05
239	MRPL34	-1.09	0.035188
240	GALK1	-1.08	0.005101
241	KANK2	-1.08	0.02491
242	DAAM2	-1.08	0.009747
243	KMT2D	-1.08	0.002113
244	HCFC1	-1.07	0.002893
245	PTBP1	-1.07	0.000734
246	DCXR	-1.07	0.010353
247	PRR12	-1.07	0.014309
248	FARSA	-1.07	0.002084
249	ZMIZ1	-1.06	0.002318
250	SPTBN5	-1.06	0.015319
251	ST3GAL4	-1.06	0.041449
252	PSRC1	-1.06	0.019303
253	CAMTA1	-1.06	0.019115
254	C16orf80	-1.06	0.029024
255	ANKRD13B	-1.06	0.002333
256	KAT5	-1.06	0.030425
257	ARRB2	-1.06	0.03037
258	RAB26	-1.05	0.002213
259	TEAD2	-1.05	0.008979
260	CDK2	-1.05	0.018909
261	BRSK1	-1.05	0.019465
262	ALYREF	-1.05	0.000547
263	TEAD4	-1.05	0.016703
264	EP400	-1.04	0.00105

Sr No	Gene Name	log2Fold Change	padj
265	WDR18	-1.04	0.014156
266	KIF18B	-1.04	0.001009
267	RPS16	-1.04	9.28E-05
268	ZNF462	-1.04	0.006494
269	SSBP4	-1.04	0.022013
270	DNMT1	-1.04	0.000468
271	NFIA	-1.04	0.049361
272	POLD1	-1.03	0.004596
273	ASPSCR1	-1.03	0.025391
274	PCGF2	-1.03	0.019465
275	MRPL9	-1.03	0.006313
276	MTRNR2L2	-1.03	0.000824
277	POLD2	-1.03	0.001258
278	RPLP0P6	-1.03	0.000633
279	HELZ	-1.02	0.004495
280	FGFR2	-1.02	0.006908
281	GNL3L	-1.02	0.021272
282	RPS2P5	-1.02	0.0001
283	PAK4	-1.02	0.033165
284	TMEM259	-1.02	0.000774
285	MRPL14	-1.02	0.014953
286	KHSRP	-1.02	0.000493
287	ZMYND19	-1.02	0.005501
288	MCM5	-1.02	0.012338
289	TONSL	-1.02	0.019849
290	SOX12	-1.01	0.006908
291	NT5C3B	-1.01	0.006134
292	MLLT1	-1.01	0.009814
293	DZIP1	-1.01	0.035677
294	AURKB	-1.01	0.003535
295	MAX	-1.01	0.015954
296	C1QBP	-1.01	0.003036
297	MYBL2	-1.01	0.006544
298	ABI2	-1.01	0.024639
299	PTPRS	-1.01	0.005145
300	HIPK2	-1.00	0.007612
301	TAF4	-1.00	0.013211
302	SMARCB1	-1.00	0.001455
303	GSE1	-1.00	0.000315
304	FASN	-1.00	0.001758
305	NUDT1	-1.00	0.008133
306	CYTH1	-1.00	0.002737
307	CDCA5	-1.00	0.019189
308	CCDC85C	-1.00	0.039

Sr No	Gene Name	log2Fold Change	padj
309	DPYSL5	-1.00	0.014948
310	USF2	-1.00	0.004493
311	POLE4	-1.00	0.045416
312	PIH1D1	-1.00	0.015336
313	SSNA1	-1.00	0.016746
314	TOMM40	-0.99	0.011417
315	AMPD3	-0.99	0.02043
316	AES	-0.99	0.002735
317	PCIF1	-0.99	0.03214
318	MMAB	-0.99	0.016291
319	PPP1R14B	-0.99	0.002511
320	MRPS34	-0.98	0.012163
321	NANOS1	-0.98	0.020232
322	CNNM1	-0.98	0.016618
323	CDT1	-0.98	0.016707
324	ACLY	-0.97	0.000737
325	THOC6	-0.97	0.040826
326	RERE	-0.97	0.003343
327	LTBP4	-0.97	0.001307
328	MCM7	-0.97	0.000718
329	POLE	-0.96	0.003869
330	TRIM8	-0.95	0.033157
331	ST3GAL2	-0.95	0.035509
332	ENO1	-0.95	0.008697
333	GPI	-0.95	0.019189
334	LRP5	-0.95	0.01185
335	EIF3G	-0.95	0.002713
336	TRRAP	-0.94	0.009814
337	TBCD	-0.94	0.001221
338	SBNO2	-0.94	0.016291
339	BCOR	-0.94	0.004341
340	PKN1	-0.94	0.005678
341	FAM65A	-0.94	0.03346
342	FBLN1	-0.94	0.002595
343	AKNA	-0.94	0.011559
344	ACTN4	-0.94	0.001214
345	40057	-0.94	0.00119
346	KIAA0930	-0.93	0.016833
347	HPDL	-0.93	0.047808
348	FOXK2	-0.93	0.010307
349	TCF7L1	-0.93	0.012338
350	WDR74	-0.93	0.013315
351	RNF213	-0.93	0.020236
352	THAP11	-0.93	0.043008

Sr No	Gene Name	log2Fold Change	padj
353	MDC1	-0.93	0.008884
354	GRAMD4	-0.92	0.015336
355	GATAD2A	-0.92	0.017504
356	EPN2	-0.92	0.025412
357	HSD17B10	-0.92	0.013414
358	ALKBH5	-0.92	0.044226
359	CHD7	-0.92	0.012295
360	TUBA4A	-0.92	0.039
361	BRD4	-0.91	0.013944
362	TECR	-0.91	0.012213
363	PPP1CA	-0.91	0.040618
364	SRC	-0.90	0.034561
365	UCK2	-0.90	0.002687
366	CREBBP	-0.90	0.018553
367	AIPL1	-0.90	0.005043
368	ARID1B	-0.90	0.010129
369	RPL13AP5	-0.90	0.022047
370	ELMSAN1	-0.90	0.019784
371	MAF1	-0.90	0.037149
372	MCM2	-0.89	0.013671
373	CIT	-0.88	0.022651
374	VPS51	-0.88	0.015837
375	COBL	-0.88	0.031294
376	UNG	-0.88	0.013562
377	MYO19	-0.88	0.015837
378	TCF20	-0.88	0.009551
379	FANCG	-0.88	0.030568
380	COX5A	-0.87	0.007211
381	LSM7	-0.87	0.04823
382	PFKP	-0.87	0.00847
383	TMEM97	-0.87	0.005652
384	TRAP1	-0.87	0.016763
385	AGAP1	-0.86	0.02539
386	LMNB2	-0.86	0.010448
387	SAMD11	-0.86	0.044901
388	BAIAP2	-0.86	0.019612
389	RANBP3	-0.86	0.040009
390	CD63	-0.86	0.008619
391	DAZAP1	-0.85	0.008884
392	APOA1BP	-0.85	0.044226
393	TTLL12	-0.85	0.021222
394	SCAF1	-0.85	0.032837
395	LPCAT1	-0.85	0.014309
396	MSI1	-0.85	0.015637

Sr No	Gene Name	log2Fold Change	padj
397	NCS1	-0.85	0.037257
398	RPS15	-0.84	0.019465
399	SPEN	-0.84	0.013409
400	RREB1	-0.83	0.045596
401	WNK2	-0.83	0.049213
402	DVL1	-0.83	0.017782
403	SETD1A	-0.83	0.043482
404	RPS11	-0.83	0.00376
405	CHAF1A	-0.83	0.041265
406	TCF3	-0.82	0.024639
407	SRCAP	-0.82	0.042143
408	E2F1	-0.82	0.02675
409	BAI2	-0.82	0.041449
410	UHRF1	-0.81	0.02186
411	BAZ2A	-0.81	0.014571
412	ADNP2	-0.81	0.041715
413	EIF5A	-0.81	0.027983
414	SIPA1L3	-0.80	0.028576
415	MRPS23	-0.80	0.037118
416	ATN1	-0.80	0.023214
417	EIF4A3	-0.80	0.015746
418	FOXM1	-0.79	0.04397
419	BSG	-0.79	0.022189
420	ATXN2L	-0.79	0.039
421	PIEZO1	-0.79	0.0352
422	FAM60A	-0.79	0.023129
423	HMGA1	-0.79	0.020789
424	GRB2	-0.79	0.040153
425	PTPRU	-0.78	0.042709
426	HGS	-0.78	0.042143
427	CDC6	-0.78	0.049948
428	TTC7B	-0.77	0.035677
429	BRD2	-0.77	0.014328
430	SNRNP70	-0.77	0.014058
431	APBA2	-0.77	0.037137
432	H3F3B	-0.76	0.012835
433	PTMS	-0.75	0.017653
434	GBA2	-0.75	0.048488
435	MDN1	-0.75	0.033472
436	RPL35	-0.74	0.010376
437	PDIA4	-0.74	0.029024
438	CD55	-0.73	0.04674
439	SMARCC2	-0.73	0.048255
440	RPL27	-0.72	0.021632

Sr No	Gene Name	log2Fold Change	padj
441	SRP68	-0.72	0.043482
442	BUB3	-0.69	0.043008
443	PKM	-0.62	0.044226
444	PPIA	0.67	0.02722
445	RPS4X	0.69	0.038643
446	EPRS	0.70	0.044901
447	XRN2	0.71	0.048956
448	COPB2	0.74	0.043647
449	GLCE	0.75	0.038685
450	OGT	0.75	0.040009
451	HSPD1	0.75	0.015261
452	GNG12	0.75	0.048057
453	40787	0.76	0.036602
454	BZW1	0.76	0.039
455	PSMA3	0.77	0.041332
456	ZNF106	0.78	0.020386
457	ACTR3	0.80	0.019038
458	RPS7	0.80	0.007529
